# QSAR Prediction Model to Search for Compounds with Selective Cytotoxicity Against Oral Cell Cancer

**DOI:** 10.3390/medicines6020045

**Published:** 2019-04-01

**Authors:** Junko Nagai, Mai Imamura, Hiroshi Sakagami, Yoshihiro Uesawa

**Affiliations:** 1 Department of Medical Molecular Informatics, Meiji Pharmaceutical University, 2-522-1 Noshio, Kiyose, Tokyo 204-8588, Japan; nagai-j@my-pharm.ac.jp (J.N.); y151038@std.my-pharm.ac.jp (M.I.); 2 Meikai University Research Institute of Odontology (M-RIO), 1-1 Keyakidai, Sakado, Saitama 350-0283, Japan; sakagami@dent.meikai.ac.jp

**Keywords:** quantitative structure-activity relationship, machine learning, random forest, natural products, tumour-specificity

## Abstract

**Background**: Anticancer drugs often have strong toxicity against tumours and normal cells. Some natural products demonstrate high tumour specificity. We have previously reported the cytotoxic activity and tumour specificity of various chemical compounds. In this study, we constructed a database of previously reported compound data and predictive models to screen a new anticancer drug. **Methods**: We collected compound data from our previous studies and built a database for analysis. Using this database, we constructed models that could predict cytotoxicity and tumour specificity using random forest method. The prediction performance was evaluated using an external validation set. **Results**: A total of 494 compounds were collected, and these activities and chemical structure data were merged as database for analysis. The structure-toxicity relationship prediction model showed higher prediction accuracy than the tumour selectivity prediction model. Descriptors with high contribution differed for tumour and normal cells. **Conclusions**: Further study is required to construct a tumour selective toxicity prediction model with higher predictive accuracy. Such a model is expected to contribute to the screening of candidate compounds for new anticancer drugs.

## 1. Introduction

Various anticancer drugs are used to treat oral cancer; however, most of these drugs also affect normal cells. Damage to normal cell induces several adverse effects, one of these is oral mucositis (OM). OM of patients who receiving cancer therapy makes difficult to eat and to deprive volition of treatment. OM is an inflammation induced by various factors such as trauma, viruses and bacterial infections, genetic factors, stress, vitamin deficiency, and chemotherapy [1,2]. The mechanism of detail is still not well known; however, toxicity to normal cells is one of the causes. In addition, many anticancer drugs are toxic to normal cells and have low selectivity for tumour cells. For these reasons, anticancer drugs which have low toxicity on normal cells are urgently needed.

Compounds which are highly tumour-specific exist in natural products. Previously, we reported cytotoxic activity against human oral squamous cell carcinoma (OSCC) cell lines and human oral normal cells using a variety of natural and synthesized organic compounds with chromone and azulene, which are present in various natural products, as the mother nucleus [3]. We have recently reported that many anticancer drugs induce keratinocyte toxicity by inducing apoptosis [4]. However, very few reports have been published [5] about the exploration of new synthetic substances that show low keratinocyte toxicity except of our studies (Table 1).

Based on the notion that similar structures have similar activity, the relationship between chemical structure and activity is referred to as the structure-activity relationship (SAR). Currently, using information about chemical structure which called “descriptor” that is structural, physicochemical and quantum chemical variety of characteristics, data were calculated and used for relation analysis. Conventionally, multiple regression analysis, which is a standard statistical approach, has been employed to analyse the relationship between the characteristic amount and activity of such drugs. Recently, machine-learning methods have been applied to such analyses due to their high prediction performance, and the quantitative structure activity relationship (QSAR) model is used to screen lead compounds in drug discovery research [6,7,8].

We have also studied the properties of compounds relative to cytotoxicity activity using QSAR analysis of compound and cytotoxic activity reported in the literature [3]. However, we could not employ high performance analysis methods due to the limited number of compounds evaluated in each study.

Thus, in this study, we gathered compound data from our previous reports and developed a database with a sufficient number of compounds to facilitate the use of a more advanced prediction method than single regression analysis. We attempted to construct a prediction model to search for compounds with high cytotoxic activity and tumour specificity score, using the collected data of cytotoxic activity of various compounds against tumour and normal cells.

To construct the prediction model, random forest (RF; one of the machine learning method) [9,10], was adopted, expecting the collection of sufficient numbers of compounds for QSAR analysis.

## 2. Materials and Methods 

### 2.1. Data Collection and Preparation

We collected our original articles published up to May 2018 (with the exception of literature reviews), and compound and cytotoxicity data were extracted from the collected articles. All OSCC and normal human oral cells were incubated at 2 × 103/96-microwell and incubated for 48 h to produce near confluent cells (approximately half of the plate covered by cells) so that cells can further grow. Cells were then treated with various conditions of samples for 48 h. Controls contains the same concentration of DMSO, and subtracted from the experimental values to correct for DMSO cytotoxicity. Relative viable cell numbers were determined by MTT method. The conditions of cytotoxic assays were the same for all experiments we have done in our previous publications [11,12,13,14,15,16,17,18,19,20,21,22,23,24,25,26,27,28,29,30,31,32,33,34,35,36,37,38,39,40,41,42,43,44,45,46,47,48,49].

Cytotoxicity data were used as a ratio of mean 50% cytotoxic concentration (CC_50_) against OSCC cell lines (HSC-2, HSC-3, and HSC-4) and human oral normal cells (human gingival fibroblast, HGF; human pulp cells, HPC; human periodontal ligament fibroblast, and HPLF), and these CC_50_ were converted to −logCC_50_ (pCC_50_), which is a negative common logarithm.

The tumour cell selective toxic index (selectivity index; SI) was defined as the ratio of the mean CC_50_ of OSCC cell lines to the mean CC_50_ of the human oral normal cells, and the SI was calculated for all individual compounds.

### 2.2. Chemical Structure Data Acquisition and Descriptor Calculation

The collected compounds were drawn using MarvinSketch 18.10.0 (ChemAxon, Budapest, Hungary) [50] and then converted to SMILES that is a form of a line notation based on graph theory, to obtain numerical data from the chemical structure.

The compound data were dealt with using the integrated computational chemistry system Molecular Operating Environment (MOE) version 2018.0101 (Chemical Computing Group Inc., Quebec, Canada) [51] as follows; salts were removed, structure optimization was calculated, and load partial charges were obtained. The structural data were converted to a 3D format by MOE using “Rebuild 3D” and structural optimization was realized by force field calculation (amber-10: EHT).

From this compound data, we calculated 2D and 3D descriptors using MOE and Dragon (version 7.0.2, Kode srl., Pisa, Italy) [52], respectively. Descriptors were treated independently by the software. Standard deviations were calculated with each descriptor; in cases where the value was zero, the descriptor was excluded. These descriptor data calculated by MOE and Dragon were merged for each compound.

### 2.3. Preparation of Data Table

The cytotoxic activity and descriptor data were merged to a data table for analysis. The compounds in this data table were checked for duplication by using SMILES. Compounds that had one SMILES to several cytotoxic activity data from different articles adopted the mean pCC_50_.

### 2.4. Construction of Prediction Models by RF

The data table was randomly split (2:1 ratio) into a training set and an external validation set [53].

Eight structure-toxicity relationship prediction models were constructed by RF using the training set. The response variables of eight prediction models were three pCC_50_ against each OSCC cell line, three pCC_50_ against each human oral normal cell, the mean pCC_50_ against OSCC cell lines (mean tumour cell), and the mean pCC_50_ against human oral normal cells (i.e., the mean normal cell).

In the same manner, a tumour cell selective toxicity prediction model in which the response variable was the SI was constructed by using RF. Construction of prediction models by RF was performed “Bootstrap Forest” [54] in statistical software JMP^®^ Pro. 13.1.0 (SAS Institute Inc., Cary, NC, USA) [55].

**Figure 1:**
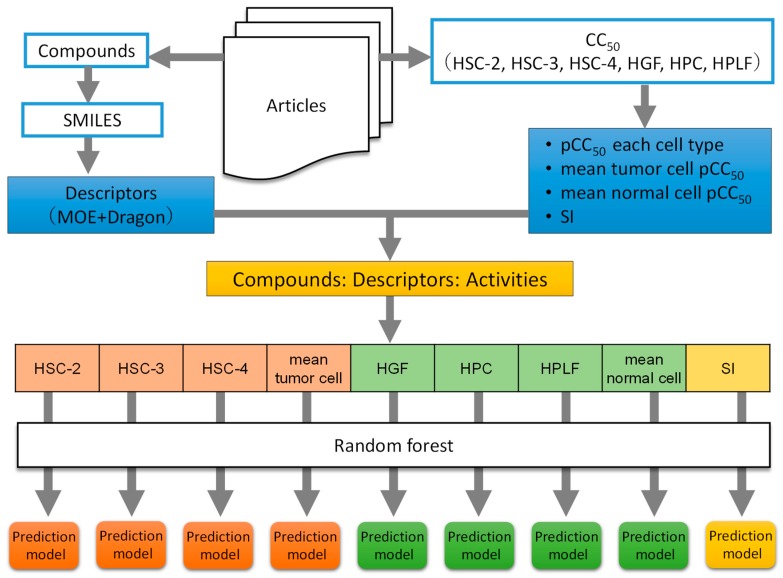
Schematic diagram of data collection and analysis.

To construct the prediction model, changing parameter settings and largest coefficient of determination prediction model that was selected. Figure 1 shows the procedures from Section 2.1, Section 2.2, Section 2.3 and Section 2.4.

## 3. Results

### 3.1. Data Collection

We obtained 498 compounds from 39 articles [11,12,13,14,15,16,17,18,19,20,21,22,23,24,25,26,27,28,29,30,31,32,33,34,35,36,37,38,39,40,41,42,43,44,45,46,47,48,49]. After eliminating duplicate compounds by SMILES, 494 compounds were analysed. Table 2 shows the articles and number of extracted compounds. These 494 compounds belong to the compound groups developed from various natural products, having skeletons shown in Table 2. SMILES data of these compounds are provided in Supplementary Materials (Table S1).

Descriptors were calculated from each software MOE and Dragon, subsequently excluded in case of the value is constant. After cleaning, 3750 descriptors were remained and used for analyses (319 descriptors calculated by MOE and 3431 descriptors calculated by Dragon).

**Figure 2:**
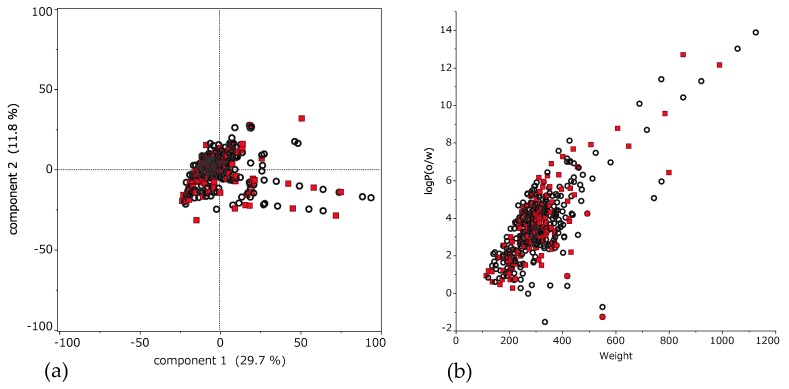
Chemical space of 494 compounds. (**a**) Applicability domain (AD) of 494 compounds. Scatter plot of principal component analysis using descriptors. The horizontal axis is the first principal component, and the vertical axis is the second principal component. These percentage are eigenvalue that represent a partition of the total variation in the multivariate sample. Each dot represents a compound; black circle is the training set and red square is the external validation set. (**b**) The horizontal axis is molecular weight (MW) and the vertical axis is octanol-water partitioning coefficient (logP). Here, each dot represents a compound; black is the training set and red is the external validation set.

Figure 2a shows applicability domain (AD). AD is the range of molecular properties or structures for which the model is considered to be applicable [56]. This scatter plot shows the result of principal component analysis using descriptors. The horizontal axis is the first principal component, and the vertical axis is the second principal component. Training set and test set compounds distribute as well balanced.

Moreover, to indicate detailed properties of these compounds, scatter plot of molecular weight (MW) and octanol-water partitioning coefficient (logP) is shown in Figure 2b. These compounds showed characteristic distribution of MW from 114.2 to 1125.8 (median 297.9); and logP from −1.53 to 13.9 (median 3.46).

### 3.2. Construction of Prediction Models by RF

Several prediction models were built by parameter turning. Here, the model that demonstrated the largest value was selected. The prediction accuracy and parameters of each model are shown in Table 3. These models were evaluated by using parameters as follows; *R*^2^, root-mean-square error (RMSE), out of bag (OOB) RMSE, maximum absolute value of the residue, mean absolute error (MAE). The OOB RMSE is computed as the square root of the sum of squared errors divided by the number of OOB observations. OOB observations are training observations that are not used to construct a tree in RF. MAE is a mean of error at a model which the value is the closer to zero indicates the model is the higher accuracy.

**Figure 3:**
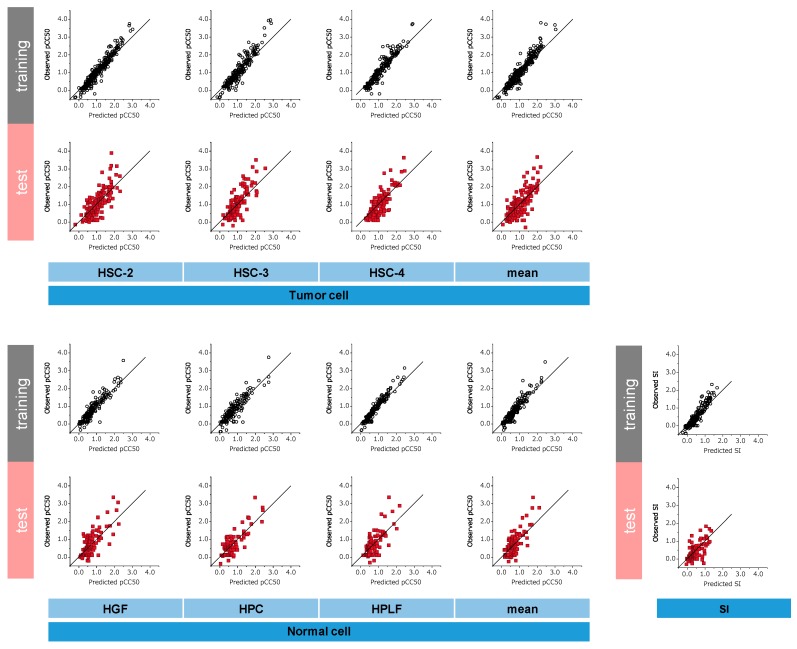
Scatter plot of training set and external validation set. In scatter plots of tumour and normal cell, the horizontal axis is the predicted pCC_50_, and the vertical axis is the observed pCC_50_ of tumour and normal cell. In scatter plot of SI, the horizontal axis is the predicted SI, and the vertical axis is the observed SI. Each dot represents a compound; black circle is the training set and red square is the external validation set.

Figure 3 shows scatter plots of each RF model obtained using the training and external validation sets, the measured pCC_50_, the predicted pCC_50_, the predicted SI, and the observed SI.

The model that demonstrated the greatest *R*^2^ value with the external validation set was the normal cell HPC model (*R*^2^ = 0.659, RMSE = 0.372), and the SI model (*R*^2^ = 0.404, RMSE = 0.340) demonstrated the smallest *R*^2^ value.

### 3.3. Large Contribution Descriptor for Prediction Model

Table 4 shows the top five contributing descriptors for the RF prediction model. Importance of descriptor were evaluated “LogWorth” in JMP^®^ Pro software. “LogWorth” is calculated as negative common logarithm of p-value. This p-value is calculated in a complex manner that takes into account the number of different ways splits can occur. This calculation is very fair compared to the unadjusted p-value [57]. In the structure-toxicity relationship prediction model, most descriptors were classified into groups representing the topological shape. Note that descriptors meaning lipophilicity were observed in the tumour cell model, and electric charge descriptors were observed in the normal cell model. Topological or 3D shape descriptors were selected in the tumour cell selective toxicity prediction model. 

## 4. Discussion

From our previous articles, 494 compounds and activity data were obtained. As reported in previous studies, compounds with higher tumour specificity than existing anticancer drugs are present among these 494 compounds. For example, in a study of 3-Styryl-2*H*-chromenes, several compounds showed higher tumour specificity than doxorubicin (at most approximately 4.8 times higher specificity) [39].

In this study, we constructed a database of seed compounds for anticancer drugs, including a sufficient number of compounds for analysis.

Regarding cell type, the structure-toxicity relationship prediction models demonstrated the maximum *R*^2^ value for cytotoxic activity against normal cells. If toxicity against normal cells can be predicted accurately, it is hoped that such prediction models can be applied to the estimation of side effects caused by cytotoxicity against normal cells, such as OM, hematotoxicity alopecia and so on. In contrast, with the training set, the *R*^2^ values were greater than 0.9. In addition, the *R*^2^ values of all structure-toxicity relationship prediction models obtained with the external validation set were greater 0.5.

We consider that the structure responsible for lipophilicity or a combination of lipophilicity and another characteristic descriptors may contribute to cytotoxic activity prediction because lipophilicity was tend to observed in the tumour cell models and not in the normal cell models. Relationship of between lipophilicity and cytotoxicity against tumour cell might be considered that penetration mechanism of compounds into tumour cell is one of the reason, however, further study is needed.

We expect that these findings will be useful relative to examining prediction models in future. In construct to structure-toxicity relationship prediction model, the *R*^2^ results of the tumour cell selective toxicity prediction models were less than 0.5.

In light of these results, further study is required to construct a tumour cell selective toxicity prediction model. With the RF method, the meaning of the top five contributing descriptors tended to differ from the structure-toxicity relationship and tumour cell selective toxicity prediction models.

These results indicate that tumour cell selective toxicity prediction is difficult to realize using the methods employed in the cytotoxic activity prediction model.

Thus, further study involving other methods, parameter tuning, and so on is required to construct a tumour cell selective toxicity prediction model with high prediction accuracy. Superior anticancer drugs require both strong cytotoxicity against tumour cells and selectivity to tumour cells, therefore cytotoxic activity prediction model is needed.

In this study, we constructed prediction models that can estimate cytotoxicity and tumour selective toxicity based on cytotoxic activity data derived from various compounds. The RF machine learning method constructed models with higher prediction accuracy.

In future, using our findings as reference, we would like to construct a high-performance prediction model that can be used to search for candidate compounds for a new anticancer drug.

## 5. Conclusions

In this study, we constructed a database of different compounds with structure and cytotoxic activity data derived from various compounds reported in previous studies. With this database, cytotoxicity and tumour cell selective toxicity prediction models were constructed by RF method. It was found that the structure-toxicity relationship prediction model tended to demonstrate greater *R*^2^ values.

In future, we expect that collecting addition compound data and investigating various model construction methods will help realize a prediction model with good prediction accuracy, which would facilitate the search for candidate compounds for anticancer drugs.

## Figures and Tables

**Table 1:** Urgency of manufacturing new anticancer drugs with low keratinocyte toxicity (data obtained from SciFinder^®^ [5] on 5 February 2019)

Search Terms	Number of Total Reports (A)	Number of Our Reports (B)	% (B/A) × 100
OSCC	8951 (100)	141	1.6
OSCC + Anticancer Drug	335 (3.70)	60	17.9
OSCC + Anticancer Drug + Tumour-Specificity	50 (0.56)	40	80.0
OSCC + Anticancer Drug + Tumour-Specificity + Newly Synthesized	2 (0.02)	2	100.0
OSCC + Anticancer Drug + Keratinocyte Toxicity	5 (0.06)	4	80.0
OSCC + anticancer drug + QSAR	27 (0.30)	25	92.6
OSCC + Anticancer Drug + QSAR+ Newly Synthesized	3 (0.03)	3	100.0

**Table 2:** Number of compounds and basic skeleton extracted from articles.

No.	Number of Compounds	Basic Skeleton	Ref.
**1**	9	Isoflavones and Isoflavanones	[9]
**2**	3	Three β-Diketones	[10]
**3**	6	Styrylchromones	[11]
**4**	3	Nocobactins NA-a, NA-b and Their Ferric Complexes	[12]
**5**	5	Betulinic Acid and Its Derivatives	[13]
**6**	2	Berberines	[14]
**7**	20	Coumarin and Its Derivatives	[15]
**8**	1	Mitomycin C, Bleomycin and Peplomycin	[16]
**9**	13	4-Trifluoromethylimidazole Derivatives	[17]
**10**	15	Phenoxazine Derivatives	[18]
**11**	7	Vitamin K_2_ Derivatives	[19]
**12**	2	4-Trifluoromethylimidazoles	[20]
**13**	10	Phenoxazines	[21]
**14**	18	Vitamin K_2_ Derivatives and Prenylalcohols	[22]
**15**	10	3-Formylchromone Derivatives	[23]
**16**	12	5-Trifluoromethyloxazole Derivatives	[24]
**17**	19	1,2,3,4-Tetrahydroisoquinoline Derivatives	[25]
**18**	19	1,2,3,4Tetrahydroisoquinoline Derivatives	[26]
**19**	12	Dihydroimidazoles	[27]
**20**	24	Tropolones	[28]
**21**	24	Trihaloacetylazulenes	[29]
**22**	22	Trihaloacetylazulene Derivatives	[30]
**23**	10	Licorice Flavonoids	[31]
**24**	4	1,2,3,4-Tetrahydroisoquinoline Derivatives	[32]
**25**	19	2-Aminotropones	[33]
**26**	12	Phenylpropanoid Amides	[34]
**27**	12	Piperic Acid Amides	[35]
**28**	15	3-Styrylchromones	[36]
**29**	16	3-Styryl-2*H*-chromenes	[37]
**30**	18	Oleoylamides	[38]
**31**	17	3-Benzylidenechromanones	[39]
**32**	18	Licorice Root Extracts	[40]
**33**	15	Chalcones	[41]
**34**	11	Piperic Acid Esters	[42]
**35**	17	Aurones	[43]
**36**	24	2-Azolylchromones	[44]
**37**	10	Cinnamic Acid Phenetyl Esters	[45]
**38**	10	Azulene Amide Derivatives	[46]
**39**	10	Alkylaminoguaiazulenes	[47]

**Table 3:** Parameters of each model by random forest

Parameters	Tumour Cells				Normal Cells				SI
	HSC-2	HSC-3	HSC-4	Mean	HGF	HPC	HPLF	Mean	
**Number of Tree**	100	300	100	100	100	100	100	100	300
**Number of Term**	952	1000	952	952	952	952	952	952	1000
**Number of Maximum Split at Tree**	100	1000	2000	2000	2000	2000	2000	2000	2000
**Minimum Node Size**	3	5	5	5	5	5	3	5	5
**Seed Value**	29	36	44	77	93	91	730	9045	124
**Number of Tree**	23	8	21	20	9	4	34	12	8
**Number of Term at a Split**	1000	1000	952	952	952	952	952	952	1000
***R*^2^** **(Training Set)**	0.904	0.847	0.868	0.876	0.862	0.815	0.908	0.858	0.817
***R*^2^** **(External Validation Set)**	0.564	0.568	0.631	0.563	0.554	0.659	0.515	0.576	0.404
**RMSE (External Validation Set)**	0.480	0.496	0.496	0.473	0.435	0.372	0.442	0.397	0.340
**OOB RMSE**	0.808	0.778	0.742	0.760	0.593	0.587	0.618	0.573	0.579
**Maximum Absolute Value of the Residue**	2.052	1.875	1.424	1.408	1.347	1.758	1.331	1.582	1.188
**Mean Absolute Error**	0.236	0.255	0.232	0.216	0.216	0.199	0.240	0.198	0.191

**Table 4:** Top five contributing descriptors for random forest prediction model.

Cell Type		Descriptor	Meaning
**Tumour Cells**	HSC-2	vsurf_D7	Lipophilicity
		vsurf_D2	Lipophilicity
		GCUT_SMR_0	Topological shape
		CATS2D_07_LL	Lipophilicity
		SpMin2_Bh(e)	Topological shape and electric state
	HSC-3	SssNH	Topological shape and electric state
		b_max1len	Topological shape
		Mor13s	3D shape and electric state
		Mor15s	3D shape and electric state
		F01[C-C]	Topological shape
	HSC-4	SpMax_L	Topological shape
		SpAD_EA(dm)	Topological shape and dipole moment
		ATSC2s	Topological shape and electric state
		vsurf_D7	Lipophilicity
		ATSC5s	Topological shape and electric state
	Mean	logP(o/w)	Lipophilicity
		vsurf_D2	Lipophilicity
		vsurf_D6	Lipophilicity
		P_VSA_ppp_L	Topological shape and lipophilicity
		SssNH	Topological shape and electric state
**Normal Cells**	HGF	GCUT_SLOGP_0	Topological shape
		F10[C-N]	Topological shape
		SssNH	Topological shape and electric state
		SpMin2_Bh(s)	Topological shape
		GCUT_SMR_0	Topological shape
	HPC	VE1_B(p)	Topological shape and polarizability
		b_max1len	Topological shape
		CATS3D_10_PL	3D shape and electric state
		h_pKb	Topological shape and electric state
		SpMin1_Bh(p)	Topological shape and polarizability
	HPLF	P_VSA_e_3	Topological shape and electric state
		GCUT_SLOGP_0	Topological shape
		F10[C-N]	Topological shape
		SssNH	Topological shape and electric state
		h_pavgQ	Topological shape and electric state
	Mean	h_pstrain	Topological shape and electric state
		h_pavgQ	Topological shape and electric state
		b_max1len	Topological shape
		SssNH	Topological shape and electric state
		F10[C-N]	Topological shape
**SI**		TDB08p	3D shape and polarizability
		F06[C-N]	Topological shape
		PEOE_VSA+1	Topological shape and electric state
		R5u+	3D shape and size
		RDF035m	3D shape and size

## Supplementary Materials

The following are available online at https://www.mdpi.com/2305-6320/6/2/45/s1, Table S1: SMILES of 494 compounds.

## Abbreviations

**HGF**	Human gingival fibroblast
**HPC**	Human pulp cell
**HPLF**	Human periodontal fibroblast
**Log P**	Octanol-water partitioning coefficient
**MOE**	Molecular Operating Environment
**OM**	Oral mucositis
**OOB**	Out-of-bag
**OSCC**	Oral squamous cell carcinoma
**pCC_50_**	−logCC_50_, a negative common logarithm
**QASR**	Quantitative structure-activity relationship
**R^2^**	Coefficient of determination
**RF**	Random forest
**RMSE**	Root-mean-square error
**SAR**	Structure-activity relationship
